# Smart Sensors and Microtechnologies in the Precision Medicine Approach against Lung Cancer

**DOI:** 10.3390/ph16071042

**Published:** 2023-07-22

**Authors:** Giulia Maria Stella, Sara Lettieri, Davide Piloni, Ilaria Ferrarotti, Fabio Perrotta, Angelo Guido Corsico, Chandra Bortolotto

**Affiliations:** 1 Department of Internal Medicine and Medical Therapeutics, University of Pavia Medical School, 27100 Pavia, Italy; s.lettieri@smatteo.pv.it (S.L.); d.piloni@smatteo.pv.it (D.P.); i.ferrarotti@smatteo.pv.it (I.F.); a.corsico@smatteo.pv.it (A.G.C.); 2 Cardiothoracic and Vascular Department, Unit of Respiratory Diseases, IRCCS Policlinico San Matteo, 27100 Pavia, Italy; 3 Department of Translational Medical Sciences, University of Campania “L. Vanvitelli”, 80131 Napoli, Italy; fabio.perrotta@unicampania.it; 4 U.O.C. Clinica Pneumologica “L. Vanvitelli”, A.O. dei Colli, Ospedale Monaldi, 80131 Napoli, Italy; 5 Department of Clinical-Surgical, Diagnostic and Paediatric Sciences, University of Pavia Medical School, 27100 Pavia, Italy; 6 Department of Diagnostic Services and Imaging, Unit of Radiology, Fondazione IRCCS Policlinico San Matteo, 27100 Pavia, Italy

**Keywords:** lung cancer, oncogenomics, semiconductors, smart sensors, personalized medicine

## Abstract

Background and rationale. The therapeutic interventions against lung cancer are currently based on a fully personalized approach to the disease with considerable improvement of patients’ outcome. Alongside continuous scientific progresses and research investments, massive technologic efforts, innovative challenges, and consolidated achievements together with research investments are at the bases of the engineering and manufacturing revolution that allows a significant gain in clinical setting. Aim and methods. The scope of this review is thus to focus, rather than on the biologic traits, on the analysis of the precision sensors and novel generation materials, as semiconductors, which are below the clinical development of personalized diagnosis and treatment. In this perspective, a careful revision and analysis of the state of the art of the literature and experimental knowledge is presented. Results. Novel materials are being used in the development of personalized diagnosis and treatment for lung cancer. Among them, semiconductors are used to analyze volatile cancer compounds and allow early disease diagnosis. Moreover, they can be used to generate MEMS which have found an application in advanced imaging techniques as well as in drug delivery devices. Conclusions. Overall, these issues represent critical issues only partially known and generally underestimated by the clinical community. These novel micro-technology-based biosensing devices, based on the use of molecules at atomic concentrations, are crucial for clinical innovation since they have allowed the recent significant advances in cancer biology deciphering as well as in disease detection and therapy. There is an urgent need to create a stronger dialogue between technologists, basic researchers, and clinicians to address all scientific and manufacturing efforts towards a real improvement in patients’ outcome. Here, great attention is focused on their application against lung cancer, from their exploitations in translational research to their application in diagnosis and treatment development, to ensure early diagnosis and better clinical outcomes.

## 1. Introduction

Microelectronics is defined as the branch of electronics that deals with miniature electronic circuits. Microelectronic devices benefit every aspect of human life, starting from devices with micro-fluidics functions first studied at the end of last century to jet printers of ink, motion sensors of pressure and humidity, microphones, and then again to the multi-sensor modules with built-in intelligence, up to the latest piezoelectric actuators [1,2,3,4,5]. Growing consolidated technology enables production in the current large volumes and the integration of different functions in the same package, with good levels of quality and acceptable costs. In this context, each sensor and its control electronics must have the necessary resources: they are put together in one package, and they have to learn and cohabit and react correctly, according to the expectations, generating data output and consistent information. That is why the hardware calibration of the sensor and the intelligent control circuit is necessary and already in the production phase of the two single objects. Then, once the two components are brought together in the final device, the calibration of the whole device is required as well. It may be necessary to also perform a calibration of the software, perhaps performed at preset intervals, even just once to ensure that the sensor is mounted in the finished system. The recent advances in microelectronics and microfluidics, together with the development of silico platforms [6,7,8], have found interesting and potentially significant applications in several aspects of cancer medicine from research to diagnosis and drug development. These novel approaches could be of help in overcoming the existing limitation in studying and designing reliable cancer preclinical models but also in reaching a more precise tumor staging and therapeutic strategy. In this perspective, lung cancer represents an ideal target to develop and potentiate the interaction between different expertise towards successful progress in disease knowledge and patient outcome. Here, we introduce the state of the art in lung cancer therapy and then aim at focusing on how novel biosensors and MEMS-based devices could contribute to knowledge, an improvement in drug design, and development of more efficient disease management in preclinical and clinical settings. In detail, we will present the current knowledge on the biologic mechanisms of lung malignant transformations and the recent advances in therapy, mainly focusing on targeted agents and immunotherapy. Then, each step of lung cancer medicine, starting from preclinical setting up to cancer diagnosis, staging, and treatment, is analyzed and the most recent advances, current, and next-future innovative applications of microtechnology are discussed (manufacturing and lithography) (Table 1). The ultimate goal of this work is, thus, to underline the need of a deeper, concrete dialogue between scientists, technologists, and clinicians to reach effective cancer patients benefits.

## 2. Genetic and Biomolecular Features of Lung Cancer

Lung tumors are characterized by an extremely high series of genomic alterations, of which a large percentage are—to date—still unknown. However, several important pathogenic gene lesions have been documented and are currently available for a routine molecular diagnosis. In NSCLC, chromosome duplications have been found in the long arm of the chromosomes 8, 17, and 19 while losses of loci are frequent in 1p, 4q, 5q, 6q, 8p, 9p, 13q, and 17p [11]. Despite the extreme molecular heterogeneity which characterizes the disease, it is evident that some genomic alterations occur frequently and non-randomly in lung cancer. The amplicons most frequently involved are the family members *MYC* (*MYCL1*, *MYCN*, and *MYC*), the members of the EGFR-mediated signaling pathway (*EGFR*, *PIK3CA*, and *KRAS*) and other genes such as *FGFR1*, *TP63*, *TERT* and the cyclins CCDN1 and CCNE1 [12]. The genetic alterations leading to neoplastic transformation are related, on one hand, to an increased activation (gain of function) of oncogenes and on the other to the inactivation of tumor suppressor genes (loss of function). Oncogene activation occurs due to the presence of somatic gene lesions (mutations, amplifications, translocations) in a heterozygous state. Inactivation of tumor suppressor genes depends on the occurrence of lesions affecting tumor suppressor sequences, such as mutations, gene deletions, silencing epigenetic. In these cases, the alterations must be present on both alleles (homozygosity) to behave as pathogenic. The main tumor suppressor genes involved in lung cancer are *TP53* (17p13.1), *RB1* (13q14.11), *CDKN2* (p16INK4 or MST1, 9p21) and several genes located in the short arm of chromosome 3 (which occur in 90% of NSCLCs). Others tumor suppressor genes identified in this large chromosomal region are: *FHIT* (3p14.2); *RASSF1* (3p21.3); *TUSC2* (FUS1, 3p21.3); *SEMA 3B* (ep21.3); *SEMA 3F* (3p21.3), *MLH1* (3p22.3); and *RARB* (3p24) [13]. There are numerous families of proto-oncogenes that contribute to the process of tumorigenesis if constitutively activated. The signaling pathways most frequently involved concern *EGFR, MYC, RAS, STAT*, and other related genes such as *PIK3CA*, *CCDN1*, and *BCL2.* Proto-oncogenes are often activated by somatic mutations and chromosomal rearrangements such as translocation and inversions that induce constitutive activation (*MYC*, *BCL2*) or create phosphorylated protein chimerics (*EML4-ALK*) [14]. Finally, micro RNAs are a highly conserved class of small coding non-RNAs (consisting of 21/25 nucleotides) that regulate gene expression in a specific way. These molecules function at the post-transcriptional level by binding to complementary sequences in the extremity 3 untranslated (3’UTR) of target messenger RNA (mRNA), which can lead to repression of protein translation, resulting in hyporegulation of protein expression. Although the models of expression and functions of microRNAs in normal cells are not fully understood, it is known that every single microRNA can target numerous transcripts, while each single gene can be the target of numerous microRNAs [15,16]. The expression of microRNAs is emerging as important in the biology of cancer and there are already numerous studies on microRNA and lung cancer yielding relevant results [17]. For example, it was demonstrated that miRNA expression has a prognostic value. Patients with lung adenocarcinoma featuring high expression of MIRN155 (21q 21.3), MIRN1, MIRN106A, MIRN93, MIRNLET7A, or MIRN145 showed a significantly worse prognosis and a reduced postoperative survival [18]. It is interesting to note that the reduced expression of two microRNAs mapped to 3p is associated with overexpression of *KRAS* and *EGFR* in NSCLC extension. In particular, *KRAS* is deregulated by the gene coding for MIRNLET7g (MIRNA let 7-g), which is located on chromosome 3p21.2 [19]. MIRN128-2 (MIRNA128b) mapped to 3p22 is, instead, able to modulate the expression of *EGFR* and therefore to interfere with the response to targeted inhibitors [20].

### 2.1. Lung Cancer Genetic Drivers: Rationale for Targeted Therapy

Among cancer genes, the gene family protein-coding tyrosine kinase (TK) function plays a central role and many of these enzymes are greatly impaired in parts of solid tumors on the basis of different molecular mechanisms. The kinases—and their phosphatase inhibitors—are the key regulators to several cellular functions, such as proliferation, migration, metabolism, differentiation, and survival, and their activation is required for cellular homeostasis; on the contrary, their aberrant activation is crucial in driving oncogenesis [21]. The tyrosine kinase (RTK) receptors represent a subclass of transmembrane cell proteins that are controlled by the presence of a specific ligand and show intrinsic kinase activity. In quiescent cells, RTK receptors are inactive; in the presence of activating lesions—as in the case of cancer—these proteins become improperly phosphorylated. The presence of somatic mutations affecting genes encoding RTK have been shown to have a causal role in many solid tumors, including those of NSCLC. Kinases tend to be altered by heterozygous missense mutations affecting the residues involved in their enzyme activity; this suggests that the mutations operate by increasing the catalytic activity of the mutated protein. This evidence also indicates that the mutated genes of the kinases act as dominant oncogenes. Additionally, TK receptors can be activated by gene translocation or due to an increased number of gene copies [22,23]. Both mechanisms have been described in NSCLC: relevant examples include the gene fusion of *ALK-EML4*, on the one hand, and the amplification of *EGFR* and *MET*, on the other. Starting from the structural characterization of RTK, several studies were then conducted to define downstream signaling pathways, this approach has led not only to new biological results but has also had important therapeutic implications. From this perspective, in recent years, anti-RTK drugs have reached clinical use and several inhibitors have been developed through a combination of research methods and strategies [24]. From the cloning of the first cDNA that encodes for an RTK (EGFR in the late 1970s) many advances in the therapy of human cancer have been achieved and different types of tumors have benefited from this knowledge. In particular, the concept that the mutated kinases mark molecular *druggable* targets has led to intense efforts to examine the mutational status of the whole kinome—a broad spectrum of human cancer types. Among RTKs, the family of receptors of Epidermal growth factor (EGF) has been extensively studied in various solid cancers, mainly in colorectal and non-small cell lung carcinomas [25]. It consists of four members: EGFR (ErbB1, HER1), ErbB2 (HER2, neu in rodents), ErbB3 (HER3), and ErbB4 (HER4). Binding of the soluble ligand to the receptor ectodomain promotes homo- and hetero-dimer formation between receptors, a crucial step for the activation of the intracellular TK domain and the subsequent phosphorylation of the C-terminal tail. The phosphotyrosine residues are then activated, either directly or via adapter proteins, as are components of downstream signaling routes, such as RAS-RAF-MEK which are mainly involved in the promotion of cell proliferation and PIK3CA-mTOR AKT which support cell motility and invasion; other critical paths activated include the STAT signal cascade and ERBB-mediated angiogenesis. As discussed above, receptor activation is mainly due to an increase in gene copy number (7p12) or an occurrence of somatic mutations [26]. The rationale for using inhibitory targeted drugs against molecular targets is based on experimental evidence that the mutated clones depend on activation of the pro-activating signaling cascade triggered by the activated protein in aberrant manner for their survival and proliferation precisely because of the gene lesion. This phenomenon is known as *oncogene addiction* [27]. Because lung cancer cells depend on the aberrant activity of a specific mutated gene (e.g., *EGFR*) to survive and proliferate, practically it is sufficient to pharmacologically inactivate it and induce growth arrest and cell death (apoptosis). According to experimental models, the inhibition of oncogenic activity by specific inhibitors can trigger an *oncogenic shock* which will eventually lead to the death of the cells [28]. This assumption is based on the biological concept that oncoproteins activate both pro-proliferative and pro-apoptotic signals: oncogene blockade creates a time window during which apoptotic signals persist in the absence of signs of survival, resulting in cell death. The most relevant clinical implication is that targeted therapy is only effective in those patients whose tumor DNA contains the alteration that makes the tumor itself sensitive to a specific drug; in other words, before subjecting patients to targeted treatments, the presence of genetic lesions that are predicted to have potential response must be verified. Although the development of anti-EGFR therapy has provided significant results, the limitations of its effectiveness quickly became evident: only about 10–20% of unscreened NSCLC can benefit from these drugs. Indeed, mechanisms of primary and acquired resistance to TK inhibitors have been discovered and studied, and novel generation inhibitors are now available [29,30,31]. Moreover, novel molecular drivers are rapidly emerging as novel actionable targets. Among them, fusions of the *RET* (rearranged during transfection) gene (10q11) rearrangements of the c-Ros oncogene 1 (6q21), neurotrophic tyrosine kinase (*NTRK*) genes, and mutations of the v-Raf murine sarcoma viral oncogene homolog B (*BRAF*) [32,33].

### 2.2. The Basis of Immunotherapy

The therapeutic strategy of patients with advanced NSCLC has changed since the introduction of immunotherapy in clinical practice thanks to the important benefits offered in terms of long-term survival, safety, and quality of life. This new treatment possibility also contributes to the creation of a valid therapeutic approach in the subgroup of patients with NSCLC featuring a *not-addicted* genotype, which represents about 80% of patients [34,35,36]. In immunotherapy, it is not the tumor, but the immune system that is targeted. The key points of this paradigm are the following: (i) immunosurveillance of tumors occurs definitely; (ii) tumors can evade the immune response; (iii) both the adaptive immune system and the innate one are involved in the recognition and elimination of neoplastic clones [37,38]. Tumor elimination implies the activation of the same recognition mechanisms used to fight pathogens [39]. It iteratively selects and promotes the generation of tumor cell variants with the capacity to survive the immune attack, through a biological process known as *immunoediting* [40,41,42]. Indeed, cancers express checkpoint proteins on their cell surface to escape detection by the immune system by sending inhibitory stimuli to attenuate immune responses; targeted inhibition of these receptors improves the response of T cells towards the cancer. The CTLA-4 (Cytotoxic T-lymphocyte-associated Antigen-4) and PD-1 (Programmed cell death protein-1) and its ligand PD-L1 are two of the numerous immune checkpoint pathways which play a critical role in the control of T cell immune responses [43,44,45]. The CTL4 molecule is induced in T cells at the time of the initial response to the antigen. The expression level of CTLA-4 depends on the extent of the report mediated by the T cell receptor (TCR). Naïve and T memory cells do not express CTLA-4 on their own surface; after the TCR is activated by encountering the antigen, CTLA-4 is transported to the cell surface. CTLA-4 works as an attenuator of the signal to maintain a constant level of T cell activation. The PD1/PDL1 pair plays a major role in the regulation of inflammatory responses due to the recognition by effector T cells the antigen in peripheral tissues. The activated T cells upregulate PD1 and continue to express it in tissues. The expression of PDL1 promotes immunosuppression. The immunosuppressive effects are related to the induction of apoptosis of activated T cells, the facilitation of anergy, and T-cell depletion [46,47,48]. Furthermore, PDL1 expression is regulated by oncogenes and miRNAs [49,50].

## 3. Lung Cancer Therapy: Principles, Rationale, and Novel Advances

Despite increasing advances in treatment, lung cancer remains a leading cause of global cancer incidence and mortality worldwide [51]. Over one hundred years ago, Paul Ehrlich defined the concept of “magic bullet” [52], meaning drugs that go straight to their designed cell-structural targets. The concept has been exploited in the challenging rationale of personalized cancer therapy. About 70% of patients present with advanced disease at the time of diagnosis, and 85% die 5 years after disease onset [53]. Appropriate therapeutic strategy depends on the histology and molecular assessment of the tumor, the stage of the disease, and the functional status of the patient. Different therapeutic options include surgery, chemotherapy, and radiant therapy in various combinations. However, the recent integration of targeted therapy and immunotherapy has changed the landscape of treatment. Surgery is the first choice in patients with early stages (I–II) non-small cell lung cancer (NSCLC). Among surgical procedures, lobectomy has the highest long-term efficacy rate because of a lower incidence of disease recurrence, than segmentectomy or wedge resection, maybe due to occult nodal metastases [54,55]. Studies comparing video-assisted techniques versus open thoracotomy show VATS gives better results with decreased pain, a shorter length of in-hospital-stay, fewer post-operative complications, and better long-term outcomes [56,57]; moreover, VATS is particularly suitable for elderly patients and, in general, for frail patients at high risk of complications with traditional surgery [58]. Stereotactic body radiation therapy (SBRT) is a valid alternative for patients affected by early stage NSCLC who have contraindications to surgery or refuse it. It uses high doses of radiation—usually from 10 to 34 Gy (grays) for each session—on a short course of time, for example 1–2 weeks [59]. Treatment is well tolerated and allows the achievement of local tumor-control rates at 90% at 3 years [60,61]. The role of adjuvant chemotherapy in early stage lung cancer has been investigated in several randomized clinical trials and smaller studies. It has been shown to improve disease-free survival (DFS) and overall survival (OS) in stages II–IIIA after radical resection [62,63,64,65], and in selected stage IB patients with a tumor size ≥4 cm. Standard regimen includes four cycles of cisplatin-based doublets (mainly vinorelbine, less frequently paclitaxel, docetaxel, gemcitabine, pemetrexed). The meta-analysis by the Lung Adjuvant Cisplatin Evaluation Collaborative Group, comparing surgery vs. surgery plus platin-based chemotherapy in 4900 NSCLC patients, confirmed the benefit of adjuvant chemotherapy with an increased survival from 64% to 67% for stage IB, from 39% to 49% for stage II, and from 26% to 39% for stage III NSCLC [66]. Although the role of neoadjuvant chemotherapy has not been as extensively explored as adjuvant chemotherapy, evidence also suggests some benefit from it in early stage lung cancer. In fact, it enables the reduction in cancer size and the prevention of metastatic spread with the advantage of being better tolerated in the pre-operative setting, as demonstrated by the NATCH trial [67]. A pooled analysis of 15 randomized clinical trials of neoadjuvant chemotherapy plus surgery vs. surgery alone, involving about 2500 patients, has shown an absolute survival improvement of 5% at 5 years and a 13% reduction in the relative risk of death [68]. On the contrary, no benefit from target therapy has been found in the context of early stage disease [69,70,71,72]. For patients with locally advanced NSCLC (stage IIIA, IIIB) not eligible for surgery and with good performance status, definitive concurrent chemotherapy with radiation therapy, followed by one year of Durvalumab maintenance treatment, is the current standard of care. One chemotherapy regimen consists of a platin-based doublet administered every 3 weeks for a total of four cycles; the radiation dose is typically 60–66 Gy in 30–33 fractions. The subsequent addiction of Durvalumab (anti-PD-L1 monoclonal antibody) as consolidation therapy, in the case of response or disease stability (if PDL-1 expression ≥1%), has been established in clinical practice [73], since the PACIFIC trial has demonstrated an increase in progression-free survival (PFS) by 11 months [74] and an increase median OS by 18.4 months [75]. Advanced lung cancer carries a poor prognosis, with a median survival rate of 8% at 5 years [76]. However, in recent years the paradigm of treatment has changed thanks to the increasing knowledge of molecular aberrations of cancer cells, which can be exploited for therapeutic purposes. Up to 69% of advanced NSCLC patients may benefit from molecular target therapy [77]. Thus, molecular testing, preferably using a broad panel-based approach, is recommended to identify potential actionable genetic alterations and to guide treatment decision [77]. Indeed, treatment with tyrosine kinase inhibitors (TKIs) of EGFR, ALK, ROS1, RET, BRAF, NTRK, MET, and KRAS is currently available and has become the standard of care in the appropriate subset of patients [78]. *EGFR* mutations are present in 10–20% of NSCLC, especially in adenocarcinomas, women, Asian populations, and never smokers [79,80]. The mutations occur within *EGFR* exons 18–21: 90% of them are deletions in exon 19 or L858R point mutations in exon 21 and both are TKIs responsive. Today gefitinib, erlotinib, afatinib, dacomitinib, and osimertinib are all FDA-approved drugs in *EGFR*-mutated NSCLCs, with third-generation TKI Osimertinib used as the first choice after the FLAURA study [81] highlighted the superiority of Osimertinib compared with first-generation TKIs in terms of OS. ALK rearrangements originate from inversions or translocations on chromosome 2 that fuse variable regions of the EML4 gene with exon 20 of the ALK gene. They are present in 2–7% of NSCLCs, especially in young, never smoker, and Asian patients [82]. Crizotinib, ceritinib, alectinib, brigatinib and, upon progression, lorlatinib are all effective options in *ALK* mutated NSCLCs [83]. Interestingly, ALK rearrangements are usually mutually exclusive with other oncogenic mutations. In exceptional cases, two mutations can coexist: in these cases, *ALK* rearrangement confers resistance to EGFR inhibitors [84]. *ROS-1* rearrangements are detected in 1–2% of NSCLCs, especially adenocarcinomas. TKIs ceritinib [85], crizotinib [86], entrectinib [87], and, upon progression, Lorlatinib [88] are all available. RET fusions are recognizable in 1–2% of NSCLSs. Several TKIs such as sunitinib, sorafenib, vandetanib, cabozantinib, alectinib, apatinib, lenvatinib, and ponatinib are currently under investigation in phase 1 or 2 trials [89]. *BRAF* V600E mutation is present in 1–2% of NSCLCs, in particular in smokers with adenocarcinomas. As in melanoma, this mutation is responsive to the combination therapy with dabrafenib and trametinib [90]. NTRK gene fusions are found in about 0.2% of NSCLCs; both entrectinib and larotrectinib are the treatment options [91]. MET mutations in exon 14 splice sites which lead to exon skipping are present in 3–4% of adenocarcinomas and provide sensitivity to capmatinib and, upon progression, crizotinib, or cabozantinib [92]. KRAS mutations, which are relatively common in NSCLCs (up to 25% of patients), although for a long time regarded as unresponsive to target therapy, may seem to benefit from MEK inhibitors (trametinib and selumetinib) when in combination with chemotherapy [93,94]. Although TKIs’ effect is impressive in the short term, the long-term efficacy of them is limited by the inevitable development of resistance under treatment, through mechanisms including somatic mutation within the target, activation of downstream effectors or alternative signaling pathways, phenotypic transformation of cancer cells, and selective pressure of drug-tolerant persistent cells leading to the establishment of drug-resistant clones [95]. In the absence of molecular actionable targets, PDL-1 level of expression determines the possible candidacy for immunotherapy, either as monotherapy or in combination with traditional chemotherapy. Several monoclonal antibodies targeting the PD receptor (nivolumab, pembrolizumab) or the PDL-1 ligand (atezolizumab, durvalumab, avelumab) have been investigated [96]. In particular, pembrolizumab is approved as a first-line therapy in the case of PDL-1 expression ≥ 50%, since it has demonstrated an improvement in tumor objective response rate (ORR), PFS, and overall survival compared to platinum-based cytotoxic therapy (Keynote-024 trial) [97]. Additionally, in a second-line setting, immunotherapy is currently established in the treatment of patients whose disease progresses after first-line cytotoxic therapy. Treatment with nivolumab has been associated with a better OSS than docetaxel in patients who had a disease progression after platinum-based chemotherapy [98]. Similar results have been observed with pembrolizumab 35 and atezolizumab 36. Not only single agents, but also combinations of them, appear promising for NSCLC management in the perspective of limiting the development of resistances and increasing efficacy. Association of PDL-1 inhibitors and anti-CTLA-4 monoclonal antibodies may result in a higher response rate [99] and several studies are ongoing [100,101]. Whereas immunotherapy may be suitable in the setting of cancers with high tumor mutation burden (TMB), another approach is necessary in case of cancers with low TMB and low immunogenicity. The induction of immunogenicity, using epigenetic modifiers, oncolytic viruses, adoptive transfer of autologous tumor-infiltrating lymphocytes, or chimaeric antigen receptor T cell therapy able to recognize and kill tumor cells, and more in general, promotion of immunosuppressive tumor microenvironment, appears promising in some preclinical and clinical models [102]. Similarly, as is seen with target therapy on long term, lung cancer cells develop mechanisms to escape immunotherapy: these include the selection of genetic alterations to create an immunosuppressive environment, the selection of neoplastic clones unable to present antigens to recognition by immune cells, and the exhaustion of T cells. Athough better tolerated than chemotherapy, immunotherapy leads to a wide spectrum immune-related adverse events (35–40% in monotherapy, 45–50% in combined therapy), of which a 4% are severe, in particular pneumonitis, colitis, hepatitis, rash, and haematologic toxicity [103]. Finally, about 20% of NSCLC patients do not express targetable molecular aberrations; in this case treatment is still limited to platinum-based chemotherapy, associated with anti-angiogenic bevacizumab in the case of no-squamous histology. Traditional chemotherapy has the bounds to exert its effect on both tumor cells and normal cells, damaging normal tissues with high replicative rate, such as the bone marrow, gastrointestinal tract, and mucous membranes.

### 3.1. Specific Challenges in Lung Cancer Therapy

Precision medicine has profoundly changed and improved the management and outcome of patients with advanced lung cancer. However, some limitations are not yet solved, and critical challenges still remain. Undoubtedly, the future of thoracic oncology is shifting towards increased molecular testing. High throughput sequencing techniques and generation of novel bioinformatic software continuously lead to an increasing number of actionable targets: the latter require biomolecular characterization and validation as drivers before leading to the design and development of specific inhibitors [104]. Comparative clinical studies are required to evaluate the efficacy of the number of agents that are constantly developed and launched in clinics. In addition, the emergence of drug-related toxicities, resistance mechanisms, and costs represent the main obstacles that will need to be addressed [105,106]. The advent of immunotherapy has further changed the therapeutic landscape of NSCLC in the absence of driver alterations. However, many patients still do not experience clinical benefit upon use of these therapies, and indeed some tumor types appear particularly resistant. In this context, the identification of genetic/epigenetic signatures able to predict the response to immunotherapy represents is still lacking [107,108] and an ambitious goal to stratify patients before selecting and addressing them to therapy. The approach will be reached by combining molecular, cellular, patient-derived xenograft, radiomic, and artificial intelligence hallmarks. Overall, significant efforts in terms of research and technology advances, as well as in expense control, will be required to provide free diagnostic and treatment services, and to reduce delays in initiating patients’ tailored therapies.

### 3.2. The Importance of Multidisciplinary Expertise

Over the past years, the improved knowledge on the biological, genetic, and molecular heterogeneity of tumors, together with the development of pharmacological technologies, has allowed the identification of molecular targets for novel therapeutic strategies. This fast process has led to overall reconsideration of the biological and genetic peculiarities that make each tumor individual. The identification of patients likely to respond to specific treatments according to the presence of relevant molecular targets (personalized medicine) requires clinical studies focused on constant and productive interaction among professionals with a significant background in the various disciplines. Thus, to generate knowledge we need to study the tumors; to study the tumors we need their representative models; and to build them with need to have access to the patients’ tumors [109,110,111]. Thus, it is clearly evident that the integration of multidisciplinary expertise and know-how is fundamental to properly manage lung cancer, and that different skills must be applied in the development of personalized diagnosis and treatment. Cancer multidisciplinary management usually refers to tumor boards, namely scheduled meetings during which cases are presented and discussed so that clinical decisions are shared [112]. The technologic revolution that has landed the clinical arena in cancer medicine [113,114] requires the growth of a complex and interdisciplinary network through which technology innovation promotes and allows the understanding, diagnosis, and treatment of advanced diseases. Overall, work in multidisciplinary teams will result in significant changes (and ultimately in cost reduction) in the diagnosis and treatment of cancer patients. 

## 4. Impact of Micro-Technologies in Cancer Medicine

**Figure 1:**
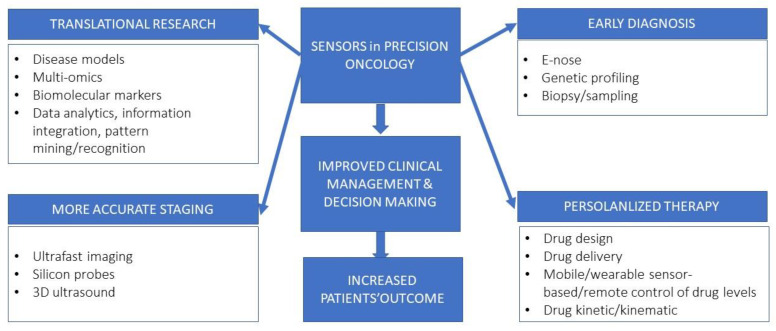
Challenges associated with the application of sensors in cancer medicine. The main aim of microtechnology application in cancer medicine is to ameliorate patients’ outcome. To reach this scope, all aspects of cancer management are involved, starting from translational research towards a more accurate system for early/molecular detection, a more precise staging based on ultrafast and 3D platforms, to advanced personalized treatments encompassing innovative devices and micropumps, and to remote controls which maintain therapeutic concentrations.

**Figure 2:**
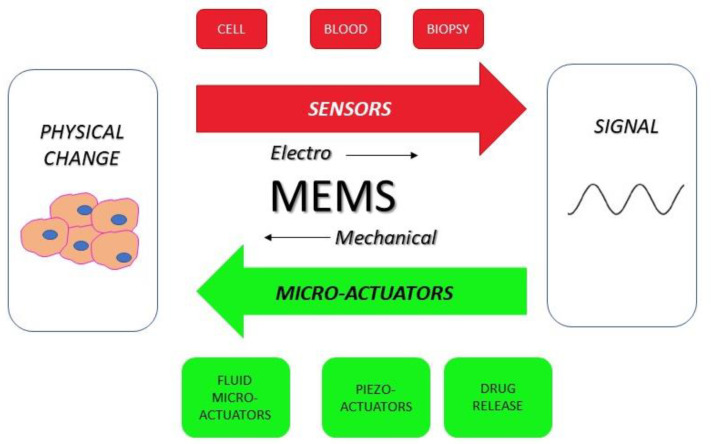
The role of MEMS in both directions and its application in cancer medicine. MEMS can be used as sensors of biophysical changes and, in this case, they generate signals that can be revealed in several biologic samples (cell, blood, or tissue). On the other hand, starting from signals detected, they can be used as actuators to modulate microfluids, pressure, and drug concentration.

Cancer is the second cause of death worldwide after cardiovascular disease, and the recent advances in diagnostic and therapeutic strategies allow a significant improvement in patients’ outcome in terms of survival and quality of life [115]. The continuous innovation in computational biology and nano-bioelectronics has paved the way to a deeper understanding of cancer genomic alteration, the surrounding microenvironment, and more efficient disease management. The main goal of clinical innovation is, on one hand, the early diagnosis cancer and, on the other, the guarantee of personalized therapy. The development of point-of-care (PoC) tests allow the patient to have a handheld test that gives the results rapidly [116,117]. Lab-on-chip (LoC) platforms permit the translation to PoC settings through process miniaturization, portability, integration, and automation [118]. LoC definition identifies a miniaturized device that integrates into a single chip one or multiple analyses, such as DNA sequencing or biochemical detection. It is clear that the application of LoC technology concerns application in cancer diagnosis, basic and translational research, and drug development (Figure 1). The technology encompasses miniaturized biosensors, high throughput analysis microarrays and microfluidics. Biosensors and micro-electromechanical systems (MEMS) have witnessed a tremendous and rapid improvement in cancer medicine due to their versatile use in diagnostic and treatment platforms. Biosensors are defined as sensitive devices which can quantitatively measure biological or chemical reactions by generating physical and/or chemical signals in proportion to the concentration of an analyte detected [119]. Based on their transducer type, biosensors can be classified as: (i) electrochemical; (ii) optical; or (iii) physical [120]. The acronym MEMS, originated in the USA, refers to Microsystems Technology in Europe and Micro Machines in Japan [121] and namely encompasses the micrometer-scaled (sized from several micrometers to several millimeters) precision devices which combine mechanical and electrical components to accomplish tasks that are normally performed by macroscopic systems [122,123] (Figure 2). MEMS are micromachining techniques in the integrated circuit industry; the main material for their manufacture is silicon [124]. LoC technology is applied in several cancer settings: high-speed genetics and genomics, proteomics with protein separation in microchips and microfluidic systems for immunoassays, determination of circulating molecules and components, and flow cytometry for single-cell rapid analysis. They are capable of performing genomics and proteomic tests on different body fluids and samples to enable point-of-care diagnostics. They are structured by using so called thermoplastics, namely high-performance polymers that can be softened and melted by the application of heat [125]. On the other hand, biosensors can be classified based on: (i) the bioreceptor (affinity or catalytic); (ii) the transducers; and (iii) their size (e.g., nanobiosensors) [126]. In cancer medicine novel biosensors are used as follows: (i) to analyze target molecules (oncogenic proteins, immune checkpoints); (ii) in transducers which can allow proper signals detection; (iii) in electronics to transduce signals through circuits and to convert them into digital form; and (iv) in displays to provide a visual images and graphs [127]. In this context, microfluidics defines a technology of manufacturing microminiaturized devices containing chambers and tunnels through which fluids can flow through for analysis; it deals with extremely small fluid volumes, such as femtoliters or lower [128]. Microfluidic biochips are currently used in cancer research to detect instance in which exosomes present chemical affinity to biochip surface related ion-exchange nanomembranes [129]. Several types of material have been used to develop biosensors, among them graphitic carbon nitride, transition metal dichalcogenides, black phosphorous, borophene, graphene, metal halides, metal oxides, metal–organic frameworks, and some polymers. Among them, early transition metal carbides and/or nitrides (MXene) feature unique hydrophilicity, electrochemical, mechanical, and optical properties and represent real-time, cost-effective sensors which are potentially useful in early cancer diagnosis [130]. With respect to lung cancer, 2-dimensional nanosheets or Ti3C2 MXene have been demonstrated to posses the ability to detect markers of cell activation, such as prostaglandin E2 (PGE2), in A549 adenocarcinoma lines. These results were more effective than those obtained through the traditional gas chromatography–mass spectrometry method, thus showing the capability of earlier detection of lung cell malignant growth. The approach relies on a multilayered structure and a large surface sensor, which based on metallic conductivity can selectively adsorb the sensor molecule 8-HOA, a marker of cyclooxygenase 2 catalyzation [131,132]. A neoplastic mass can be detected, at least at routine imaging screening, once it has reached a diameter of at least 0.5/1 cm [133,134]; however, noninvasive blood tests based on circulating tumor DNA and epigenetic traits can allow earlier diagnosis [135,136,137]. This approach has been widely implemented and validated for lung cancer as well for a review, see [138,139,140,141,142]. Notably, lung cancer marker detection can be more efficiently reached through the so-called semiconductor quantum dots (QDs), namely nanoparticles featuring stable and intense fluorescence which are now employed to expand the in vitro analysis of blood and tissue samples, as well as molecular imaging [143]. QDs are made of inorganic semiconductors, containing electrical charge carriers, namely negatively charged electrons and positively charged holes. Their excitation can return to its prior state through the emission of photons that can be revealed by fluorescence in the spectrum from ultraviolet through to visible and into the infrared rays [144,145]. This property is crucial for using QDs as sensors to detect biomarkers since it allows for highly sensitive detection in comparison with organic dyes and fluorescent proteins. Moreover, QDs probes can be used to simultaneously track multiple markers in tissue biopsies and live organs. In this case, hydrophobic dots must be made water soluble to increase target specificity. In lung cancer, graphene QDs have been evaluated in photolytic and hyperthermia therapy as well as drug carriers for targeted delivery in in vitro models [146]. Graphene is defined as a two-dimensional crystal composed of monolayers of carbon atoms arranged in a honeycombed network with six-membered rings [147]. Within the past two decades, microreactor technology has emerged with promising devices for modelling and studying cancer. They are versatile sub-millimeter tools which perform (bio)chemical transformations [148]. In medicine, their application varies between biotechnology, diagnostics, and pharmaceutical industry, making a LoC approach possible. The microreactor array system essentially constitutes of a microarray and a sealing membrane, with two facing surfaces. A reagent gap is added through a specific injector and an applicator provides fluid between the microarray and the membrane. Finally, microdetectors assure proper reaction reading [149]. With respect to lung cancer, microreactors have been studied as potential noninvasive detection systems in the analysis of exhaled breath and DNA sequencing [15].

## 5. Micro-Technologies in Preclinical Setting

Traditional preclinical research in cancer medicine has been performed in in vitro (cell cultures, 2D) and in vivo models. Functional analysis of the newly discovered targets and drug screening studies can be now reached through the generation of patient-derived xeno-patients (PDX) directly obtained from bioptic/resected primary and matched metastatic tissue. These models are generated by implanting tumor cells or fragments from patients with cancer into a transplant-compliant mouse host. PDXs have been widely developed in lung cancer since they are able to maintain tumor heterogeneity, genetic drivers, and some traits of the surrounding microenvironment [150,151,152,153]. Most recent advances in in vitro 3D culture technologies, such as organoids, have paved the way for cultures which highly resemble human cancer. However, several limitations affect the reliability of PDXs [154], and organoids too [155]. The most significant issue is related to the poor concordance between animal models and corresponding human cancers, with a subsequent limitation in clinical translation [156,157,158]. The novel organ-on-a-chip (OoC) technique emerged as a potentially useful model to recapitulate tumor growth. They are essentially defined as engineered or natural miniature tissues or cell cultures grown inside microfluidic chips [159,160]. The chips are functionalized to control cell microenvironments and maintain tissue-specific functions; therefore, this approach can guarantee a higher adherence to the Three Rs principle in research, which refers to the replacement, reduction, and refinement of animals used [161]. They are fabricated using micromanufacturing tools, such as photolithographic etching adapted from computer microchip technology, and enable the detection of living cells in their natural tissue environment [162,163]. Recent advances encompass the generation of inkjet-printed, micron-thick, and three-layered tissues, more physiologically like the human lung [164,165]. Moreover, microfluidic systems can simulate the complex cell metabolism and drug pharmacokinetics; through these models the evaluation of many types of human diseases can be achieved [166]. The lung was among the first model developed as OoC by Huh and colleagues. They developed a tissue–tissue interface of pneumocytes and endothelial cells with a porous membrane in a biosystem which recalled the alveolar-capillary membrane and the mechanical stretch of alveolar ventilation [167,168]. The technology of OoC has been widely exploited in cancer, and mainly in NSCLC, to generate reliable models for the study of tumor–stroma interaction, tumor mechanisms of growth and spreading, and the management and monitoring of responses to targeted therapies [169]. In 2018 Yang et al. developed a chip carrying a co-culture of human lung adenocarcinoma cells (A549) and human fetal lung fibroblasts (HFL1) to evaluate their response to the anti-EGFR inhibitor gefitinib [170]. Park and colleagues generated a 3D vascularized lung cancer-on-a-chip model derived from decellularized lung extracellular matrix-derived hydrogel, carrying growth and angiogenic factors, and spheroids derived from A549 cells, certified endothelial cell line isolated from umbilical cord (HUVECs), and human lung fibroblasts [171]. With a similar rationale, Khalid et al. generated a multi-sensor lung cancer-on-chip platform derived from NCI-H1437 metastatic adenocarcinoma cell lines to perform drug screening tests based on trans-epithelial electrical impedance analysis. The tool was able to measure the cytotoxicity of different concentrations of the standard chemotherapy agents doxorubicin, and docetaxel [172]. More recently a microfluidic device was developed with channels aimed at activating the immune cell environment in blood vessels to specifically test immunotherapy agents [173]. Overall, the authors demonstrated, based on drug screening analysis, that the OoC model is a more efficient and reliable tool than the conventional in vitro assays and represents a novel step towards fully personalized cancer medicine. Lung cancer-on-a-chip technology has been already applied in liquid biopsy, mainly in isolation of biomarkers from circulating tumor cells and circulating tumor DNA [174], with potentially relevant implication in early disease diagnosis and the detection of resistance to targeted therapies.

## 6. Frontier Technologies in Lung Cancer Clinical Management

### 6.1. Mutational Profiling Analysis

Cancer is a disease of genes, driven by the occurrence of gain-of-function mutations in oncogenes and loss-of-function changes in tumor suppressor genes, which results in inappropriate cell survival and proliferation [175]. Thus, somatic genetic alterations affecting the sequences of specific genes are, on one hand, the hallmark of the disease, but can also be used to classify cancers and are the best predictors of response to cancer targeted therapies [176]. Over the past two decades, an increasing number of mutations have been discovered; functionally validated and targeted therapies have landed in the clinical arena and significantly improved quality of life and survival of cancer patients [177]. The histologic and biologic heterogeneity that characterizes lung cancer has represented a main obstacle towards the development of efficient personalized treatments. Nonetheless, a rapidly growing understanding of NSCLC has led to the approval of several inhibitors against driving mutated proteins, such as the EGFR family (ERBB-1, ERBB-2), ALK, ROS1, MET, RET, NTRK, and RAF [178]. The identification and quantification of activating mutations in lung cancer is now mandatory to define therapy not only in advanced cases but also in neoadjuvant settings [97,179,180,181,182,183]. This success has been made possible in large by technological advances in sequencing which allow for massively parallel analysis. From the start of in silico DNA amplification with polymerase chain reaction (PCR) [184], digital PCR and next-generation sequencing techniques now allow the detection and quantification of mutations in transformed tissue and circulating tumor DNA fragments [185,186,187]. Advanced technology for DNA amplification and analysis regards the generation of different devices through photolithographic processes with improved hybridization capabilities in silico. In these microsystems, PCR is performed in channels embedded in silicon, with the great advantage of miniaturizing volumes and offering high surface to volume ratios, optimizing thermal performances, and assuring equivalent performances to the best thermocyclers available today. Metal oxide and metal sulfide-base nanoparticles assure accuracy and rapid diagnosis [188]. Different LoC approaches, based on MEMS technology, are designed up to today. The amplification system consists of a micro-chamber reactor with a hybrid silicon-polymer structure and the temperature control systems are implemented with microheaters integrated on a silicon substrate. For instance, on the silicon microsystem, the process for grafting DNA probes to electrodes can occur through electrochemical polymerization of a pyrrole group [189] or highly performant composition of two PCR microreactors and a microarray hybridization microchamber, fluidically connected by buried bypass [190]. In addition, [117]. Indeed, based on redox potential, metallic nanoparticles can act as a tracer. Nanoparticles, namely silver ones, featuring stronger affinity to pair DNA through magnetic beads and aggregates (rather than base pair), should be revealed by electrochemical signals [191]. Overall, these approaches assure acceleration of the amplification process, costs decrease as a consequence of lower volume of reagents used, and increased accuracy related to the automation of processes. It should also be noted that oncoprotein detection can be performed in LoC approach through microfluidic immuno-biochips, which exploit specific antigen-antibody reactions [192,193]. It has been observed that nanoparticles associated with biochips improve the sensitivity of system-immune biochips and that graphene nanosheets display better performance than other materials due to their high electrical and optical conductivity [194,195]. Notably, innovative photoactive materials have been used to detect miRNAs, such as let-7, both in cancer cells and in patients’ blood through use of a sensor constructed of carbon paste amended with silver nanoparticles and extracted propolis; it presented a very low detection limit of (10^−3^ femtomolar) [196].

### 6.2. MEMS Sensor-Based Electronic Nose

Exhaled breath analysis is widely known to have the potential in the early diagnosis of several pulmonary diseases since the emission of certain molecules and the composition of exhaled pattern reflects the metabolic alteration deep in the lungs [197]. The most relevant example regards the analysis of exhaled nitric oxide (FeNO) diagnosis in the management and phenotypization of asthma [198]. The analysis of exhaled condensate has also been investigated to detect and monitor lung cancer. In the last fifty years, several types of chemical sensor have been developed based on the concept which recalls the bio mechanical and biophysical features of the mammalian nose. First, tools were characterized by several limitations; the first e-nose was developed in 1994 and carried an array of chemical sensors and a proper pattern recognition system to discriminate gases [199]. From this date, a number of sensor types have been developed such as optical gas sensors, polymers, metal oxide semiconductors (MOS), field effect transistors (FET), catalytic, electrochemical, piezoelectric, and micro-electro-mechanical system (MEMS)-based sensors [200,201]. Other efficient techniques to detect VOCs encompass gas chromatography, gas chromatography-mass spectroscopy, proton transfer reaction-mass spectrometry, selected ion flow tube technique-mass spectrometry, ion mobility spectrometry, and high-pressure liquid chromatography. In an e-nose system, each signal analyzed is converted into an electrical signal pattern; then it is converted into higher or lower dimensions depending on the chemicals of interest and classified according to the following categories: graphical, multivariate, and neural networks. Finally, sensors’ responses are available as a bar plot or polar plot [202]. With respect to VOCs, lists of molecules that could be tested have been available since the end of the 1980s and have progressively enriched and enclosed the following molecules: benzene, toluene, styrene, decane, isobutane, methanol, ethanol, acetone, pentane, isoprene, isopropanol dimethylsulfide, 2-methyldodecane, 2-tridecanone, 2-pentadecanone, eicosane, SCC-2-decanone, 2-hendecanone, 2-methylnaphthalene, nonadecane, eicosane carbon disulfide, aldehyde-butanal, formaldehyde, acetaldehyde, pentanal, hexanal, and octanal. However, very few of these, mainly oxidative stress products such as alkanes and monomethylated alkanes, have been demonstrated to be detectable in the breath of patients with lung cancer [203,204]. The application of *-omics* technology to e-nose allowed a wider development of this non-invasive strategy to identify lung cancer patients and differentiate them from smoker subjects [205]. Great advantages in this context have been reached with the development of MEMS electronic nose (e-nose) for monitoring disease-specific volatiles in exhaled breath [206,207]. It is possible to evaluate products and altered proteins in consequence of genetic mutations. They are related to hypoxia and microenvironmental chances in consequence to metastatic activation, such as re-regulation of the expression cytochrome p450, reactive oxygen species (ROS) [208], and oncogenic protein such as mutated-EGFR [209]. Thus, the new generation of electronic devices are portable and relatively inexpensive tools that have been validated to detect VOCs in a clinical setting. The artificial olfactory sensor device Cyranose 320 (Smith’s Detection, Edgewood, MD, USA) correctly detected lung cancer patients from subjects affected by other respiratory diseases according to logistic regression analysis [210]. In a similar fashion a number of different tools, such as the ENS Mk 3 [211], nanoscale- and semiconductor-based noses, have been tested to detect lung cancer early [9]. A recent multicenter trial evaluating 575 subjects (376: training set, 199: validation set) demonstrated that matching clinical data with the exhaled breath, analyzed by handheld electronic nose device featuring an array of three metal-oxide sensors, enables real-time breath analysis (aeoNose) [212,213] and assured the proper ability to diagnose lung cancer [214]. Finally, recent reports suggest the role of artificial intelligence enabled medical sensors [215]. The main advantages of e-nose are related to the technical features [216]; however, limited data were available until now and validation in a clinical setting is not fully reached.

### 6.3. Diagnostic Applications

#### 6.3.1. Three-Dimensional Ultrasound

The novel generation of three-dimensional (3D) ultrasound (US) represents a diagnostic methodology that can open new routes for modern medicine. For instance, it can be used to simplify neuro-navigation, namely, the meticulous non-invasive exploration of images of pathology-affected brain areas on which it is necessary to intervene surgically. Moreover, 3D ultrasounds are very useful in driving the tools of the surgeon, thus avoiding many open operations in favor of less invasive techniques. The technology is based on an overall strategy of development of new generation transducers integrated with the control electronics, i.e., the elements that emit the signals add ultrasound towards the object/organ that should be examined in order to detect the response. Ordinary traditional ultrasound probes use specific piezoelectric ceramics which, when suitably excited, emit ultrasound waves (waves characterized by frequencies of 20,000 Hz or higher, which are inaudible to the human ear) to scan tissue and biological systems, analyze them, and reconstruct a faithful 2D image [217]. To be able to rebuild and view ultrasound images in 3D, silicon-based MEMS ultrasonic sensors are directly integrated into complementary metal–oxide–semiconductor-based control and processing electronics. The most effective disposition of transducers into the probes is their recalling a clockwise pattern and counterclockwise spirals organized according to the Fibonacci series [218]. Thin film piezoelectric actuators for ultrasonic applications could be the basis of new fingerprint sensors or new tools capable of seeing the objects to which they come into contact with and recreating a precise 3D image of them. Soon, 3D ultrasound of the future may no longer compromise a weighty hospital machine and it will be possible to manage a pocket ultrasound probe, or nearly so, which connects to the USB socket of a PC equipped with a special software for processing with immense benefits and the potential to diagnose in on site, outpatient, remotest places [219]. Notably, MEMS-based transducers and microphones can be also applied to endoscopes for diagnostic procedures [220].

#### 6.3.2. Silicon Probes

Tomographic ultrasonic and photoacoustic (optoacoustic) techniques are novel imaging tools which are under development and have presented great innovation in the recent years [221,222]. Nevertheless, most advancements involve the development of different types of transducers, such as piezoelectric, micro-machined based, and optical sensors [223,224]. Piezoelectric transducers can be defined as transducers able to convert any form of pressure or mechanical force into electrical energy and vice versa [225]. Piezoelectric MEMS ultrasound transducers present a thin-film piezoelectric membrane sealed between two electrodes a passive elastic layer and a substrate; a silicon-on-insulator technology is mainly used for substrate generation made of a layer of silicon buried oxide between a silicon layer and a silicon support [226]. The use of silicon for the transducer has also facilitated the coupling with the microelectronic control circuit, also obviously made of silicon. The main gains related to the use of novel piezoelectric devices are (i) low levels of background noise; (ii) reduction in the power consumption; (iii) multifunctionality; and iv batch production [227,228]. Ultrasound-on-chip, the first to be cleared by the Food and Drug Administration for 13 indications, comprises a two-dimensional array of silicon-based microelectromechanical systems (MEMS) ultrasonic sensors directly integrated into complementary metal–oxide–semiconductor-based control and processing electronics which enable an inexpensive whole-body imaging probe [229,230,231].

#### 6.3.3. Ultrafast Imaging

Echography traditionally refers to pulse focused ultrasound waves which are reflected as echoes from the tissue/organ investigated. Novel piezoelectric micromachined ultrasound transducers can emit a band of thousands of Hertz in the so-called plane waveform of sinusoidal excitation [232,233]. The plane waveform is really a big step ahead because it guarantees the acquisition of the entire image area in a single transmission, thus allowing images of clearly superior quality in ultrafast time [234,235,236]. This approach allows wider uses of medical ultrasound such as in transient elastography, blood flow evaluation, functional imaging of the brain, ultrasound localization microscopy, and setting of contrast flow [237,238]. The main disadvantages of this technique are, on one hand, the lack of focus, and, on the other, the large amount of data produced and received to reconstruct the image. To overcome redundant data, different systems of analysis can be combined to plane wave, mainly [239]: (i) Sparse arrays on the reception. In this case, some elements without significant value can be ignored and appear as zeros on the matrix form [240]. (ii) Migration which implies the reconstruction and analysis of existing geometric relations on the reflection echoes in relation to different acoustic impedances [241]. Overall, the advantages of plane wave imaging in cancer make it possible to evaluate tissue mechanical properties, and to deeper characterize the neoplastic mass and its interaction with the surrounding stroma.

#### 6.3.4. Therapeutic Implications

Ultrasounds (USs) can produce two types of biological effect, namely thermal and nonthermal. Intense USs induce relevant local variation of medium density and pressure and, consequently, molecules are forced to move rapidly. This phenomenon induces substances to overheat. On the other hand, in liquids and in some tissues, it induces the phenomenon of cavitation, namely the formation of bubbles derived from the mechanical fracture of the liquid where the pressure is reduced [242]. This effect is, for instance, can be exploited to generate small droplets for inhalators that enable the drug to reach the alveoli [243,244,245,246]. During aerosol generation, the cavitation bubbles contribute to the ejection of droplets from the liquid and allow generation of effective drug delivery [247]. Moreover, ultrasound combined with microbubble-mediated sonoporation has found application in medicine, mainly in intracellular gene therapy and drug release [222]. Indeed, the formation of the pores on the cell membrane can allow the transfer of DNA/RNA through them. Interestingly, exogenous expression of the pro-apoptotic gene *TRAIL* and *p53* in hepatocarcinoma cell lines has been reported with promising results [248] and, in a similar cell model, four complementary miRNAs (miRNA-100/miRNA-122/antimiRNA-10b/antimiRNA-21) encapsulated in a biodegradable PLGA-PEG nanoparticle have been delivered by sonoporation and showed a synergistic effect with conventional chemo (doxorubicin) [249]. Sonoporization has also found application in tissue engineering [250]. The technology has been used to demonstrate that lung stem/progenitor cells together with gelatin microbubble-scaffolds act by inducing neoangiogenic and pneumocyte differentiation, reconstructing an alveoli-like structure [251]. Moreover, it can be exploited for drug administration. The main results are derived from experiments which tested drugs effects on pancreatic and liver cancer cell lines and sferoids. The results of drug screening on doxorubicin (DOX), 5-fluorouracil (5-FU), and paclitaxel (PTX) in A549 cells demonstrate that this approach is very promising, mainly in 3D cultures [252]. Similarly, it has shown the ability to enhance the effectiveness of standard chemotherapy in pancreatic cancer [253]. Targeted therapies can also be efficiently delivered by microbubble related ultrasound as demonstrated for anti-EGFR in gingival squamous cell carcinoma [254].

## 7. Pharmaceutical Manufacturing and Drug Delivery

The generation of bio-MEMS requires manufacturing to be performed according to GMP (good manufacturing practice) and is performed in dedicated cleanroom areas to avoid contamination by moisture droplets or sub-micron particles, like dust, chemical vapors, and pathogens. The Bio-MEMS and biosensor development and market are expected to increase in the future, mainly after the COVID-19 pandemic [255,256]. Therefore, different strategies are required to micro-assemble each subsystem in joining polymer substrates and package of miniature medical devices [257]. Miniaturization is the key for faster, smarter, and more efficient devices. The most relevant application of MEMS in pharmacology manufacturing involves the design and development of drug delivery technology. The latter concerns a system to carry drugs and molecules into or throughout the body. With respect to cancer, several strategies have been investigated in recent decades with the main objective of reducing the systemic toxicity of chemotherapy and also increase the therapeutic effect on the malignant mass [258,259,260,261]. Lung cancer is a disease difficult to locally treat, with high rates of disease recurrence even after surgery is performed with curative intent [262,263]. Micro and nanoparticles loaded with chemotherapy agents have been tested in several preclinical and clinical trials [264,265,266]. A major limitation in conventional drug release regards long-term treatments, complex schedules, vehicling novel biologic and immunoactive agents, gene therapy, and, in case of lung cancer, particularly in the case of cancer-associated pleural effusion, the facilitation of effective doses to the peripheral lung and the pleural space. Most consolidated approaches involve aerosol delivery [267,268,269,270,271] and cell therapy [272,273,274]. Although a full description of the novel strategies for drug delivery in lung cancer goes beyond the scope of this review, it should be remarked that the emergence of microsensors and systems has had a fundamental upgrade this challenge. MEMS drug delivery devices are used as single and multiple reservoirs in which drugs are loaded. MEMS based implantable systems for controlled drug release in subcutaneous reservoirs are used in clinical settings [275]. Biocompatibility, bonding, and optical transparency are required features [276,277]. Different strategies have been developed regarding bio-MEMS for drug delivery. Different materials are used for reservoirs and different drug formulations have been tested [278]. In this kind of device, small and compact micropumps are incorporated into the reservoir to achieve a precise drug release. Micropumps are continuously rearranged to improve their performance in drug dosing and timing [279]. Mechanical micropumps, namely displacement micropumps, use oscillating diaphragms to pump a fluid by applying pressure generated by piezoelectric, magnetic, and material phase change [280]. Non-mechanical micropumps exert their forces through electrochemical reactions, generation of acoustic bubbles, and acoustically oscillating solid structure [281]. Implantable multi-reservoir-based devices present potential advantages for anticancer therapy development since different chemotherapeutic agents can be released passively to test their tumor sensitivity in *vivo* . This approach has been tested for several anticancer drugs such as doxorubicin, sunitinib, lapatinib, antibody cetuximab, dasatinib, gemcitabine, paclitaxel, and cisplatin [282]. Transdermal drug delivery systems (needle-based and needle-free injectors) have received great attention with the improvement of chemical enhancers and/or physical devices that enable skin permeability and the penetration of molecules. The electrical power is guaranteed by internal Li-ion micro-batteries and, more recently, by thin film micro-batteries with reduced space occupation [283]. Overall, the main advantages of bio-MEMS in drug delivery involves their ability in personalizing drug release, mainly in chronic treatments. Several strategies have been implemented to increase power efficiency: drug delivery pumps [284], low-power active components (valves, actuators) [285,286], and batteries with increased life. Microneedles can be integrated into either reservoir-type or matrix-type patches, for transdermal drug release. Other approaches involve, for example, the use of gelatin methacryloyl (GelMA) in the lithography of biodegradable microneedles. This kind of device, loaded with doxorubicin, has been used in melanoma cell lines and shows promising results [287,288]. Polymeric microneedle-based devices have been demonstrated to guarantee the prolonged release of doxorubicin for cancer treatment [289]. An analogous system has also been proposed as an adjunctive approach in solid tumor after thermal ablation. Authors reported a pharmacokinetic/pharmacodynamic model in which a doxorubicin-loaded implant (transmitter) was inserted into a solid tumor (channel) with the ultimate goal of optimizing disease control [290]. Similarly, the delivery of STAT3 siRNA by dissolving polyethylenimine microneedles has been proposed against melanoma [291].

## 8. Conclusions

The implication of this review is eminently clinical: it not only focuses on the state of-the art of microtechnology, but it also underlines how the biological and medical application of novel sensors and MEMS has rapidly revolutionized the management of cancer patients with a significant impact on their outcome. Moreover, the novelty regarding the therapeutic options ranges from innovative biosensors and implants to the possibility of working with atomic concentrations of drugs, thus avoiding systemic toxicity and increasing precise cancer-specific drug release.

Novel biosensors and microsystems have created new paradigm in medicine, most importantly in oncology. This technological advancement not only pushes research achievements but also impacts on cancer patients’ outcomes and quality of life. It is emerging that interdisciplinary work and know-how is fundamental to effectively personalize cancer diagnosis, staging, and treatments. This issue is reflected in the role of clinicians who need to have multilevel competences. Finally, it should be noted that novel materials, among which semiconductors, appear to be safe for workers and are not clearly associated with a specific risk of cancer development [292,293,294]; however, adequate control exposure and monitoring programs should be applied by global agencies.

## Figures and Tables

**Table 1:** Main ongoing trials exploiting smart biosensors and devices and MEMS in the different steps of lung cancer management.

Study Focus	Device/Sensor	Study Scope	Reference
Preclinical	Patients’ derived organoids based on 3D nanomatrices and 3D tumors Microfluidic device Microfluidic device	Evaluation of tumor functional vasculature Purification of circulating tumor cells Analysis of EGFR-mutated circulating tumor cells	NCT04826913 NCT04957602 NCT01193829
Diagnosis	MEMS e-nose MEMS e-nose Acoustic sensors array (e-stethoscope) Wireless palpatory Electrosensing antibody probing system	Mutation detection Analysis of cancerogenic VOCs after surgery for cancer Mapping chest sound propagation Detection of subpleural tumors To improve costs and save time for mutation detection	[9] NCT0803137 NCT03043898 NCT03521615 NCT01359436
Disease Monitoring	Mobile sensor technology MEMS mixed approach	To help in assessing symptoms, response to therapy and quality of life Adherence to therapy and persistence in relation to clinical outcome	NCT04465214 NCT 04347161
Treatment	MEMS magnetic field sensor-based Artificial intelligence/Machine learning	Radiotherapy: more accurate breathing signal graph with lower measurement error and higher spatial resolution than conventional Prediction of efficacy of immunotherapy	[10] NCT05537922

## Data Availability

Data is contained within the article.
